# Medication adherence interventions for South Asian populations with cardiovascular disease: A systematic review and narrative synthesis

**DOI:** 10.1371/journal.pgph.0006809

**Published:** 2026-08-12

**Authors:** Hiranmayi Madabhushi Hari, Anna Robinson-Barella, Andrew Husband

**Affiliations:** 1 School of Pharmacy, Newcastle University, Newcastle Upon Tyne, United Kingdom; 2 Newcastle NIHR Patient Safety Research Collaboration (PSRC), Newcastle University, Newcastle Upon Tyne, United Kingdom; BRAC University Institute of Educational Development, BANGLADESH

## Abstract

South Asian populations experience a disproportionate burden of cardiovascular disease (CVD) globally, with evidence consistently highlighting suboptimal medication adherence across both regional and diaspora settings. While previous research has primarily identified socio-cultural barriers to adherence, there is a critical need to analyse the effectiveness and feasibility of interventional solutions to improve medication adherence in this population. This systematic review synthesised evidence on the reported effectiveness and feasibility of such interventions from 29 studies published between 2004 and 2025. Data from India, Pakistan, Nepal, Sri Lanka, Bangladesh and diaspora settings (*e.g.,* United Kingdom), were analysed using a narrative synthesis approach. Twenty-six studies reported statistically significant improvements in adherence (*p* < 0.05). The interventions identified spanned seven themes: behavioural/educational strategies, task-shifting models, mHealth tools, regimen simplification, socio-cultural determinants, measurement of adherence and cultural inclusion practices. The findings highlight a clear ‘sustainability gap’, where short-term technological or educational bursts often fail to maintain long-term adherence without sustained human interaction or professional follow-up. Furthermore, while pharmacological simplification *via* polypills, mHealth reinforcement and task-shifting models are effective and feasible, there remains a critical paucity of culturally tailored, faith-sensitive interventions designed to address non-adherence driven by socio-cultural beliefs. Future research must transition toward robust, context-specific socio-cultural adaptation by incorporating objective adherence measures and individualised care models that engage faith-based factors. Ultimately, achieving equitable cardiovascular outcomes in this population requires a multi-level approach that integrates professional reinforcement with scalable technology to ensure long-term sustainability beyond short-term trial effects.

## Introduction

Cardiovascular disease (CVD) is the leading cause of mortality worldwide, accounting for nearly one-third of all global deaths each year.[1,2] In South Asia, comprising India, Pakistan, Bangladesh, Sri Lanka, Nepal, Bhutan, and the Maldives, the burden of CVD has increased in recent decades, with earlier disease onset and outcomes often worse than those observed in higher-income settings.[3,4]

Medication adherence is central to the long-term management and secondary prevention of CVD. Non-adherence to prescribed cardiovascular medications, such as antihypertensives, antiplatelets and lipid-lowering therapies substantially increases the risk of recurrent events, hospitalisation and mortality.[5] However, evidence from South Asian populations suggests that adherence remains poor, with self-reported adherence estimates largely derived from validated questionnaires and patient interviews, typically ranging between 30% and 70%, varying by disease context and healthcare setting.[4,6–8]Reported barriers to adherence remain multifactorial and have been attributed to factors including costs of medication, gaps in patient education and health literacy, cultural perceptions about chronic therapy and limited follow-up and counselling opportunities within overburdened health systems.[5,8–10] Recent qualitative evidence [11,12] has highlighted lower levels of medication adherence amongst those from ethnically minoritised groups, including people from South Asian populations living within the United Kingdom, United States, Canada, Australia, Qatar and New Zealand. These studies have demonstrated that adherence behaviours can be intersectional, shaped by an interplay of cultural, ethnic, linguistic and systemic factors.

Globally, numerous strategies have been developed with the intention of improving medication adherence for people with CVD; these have ranged from educational and behavioural theory-based interventions [13,14] to pharmacist-led [15], multifaceted approaches.[16–18] However, many of these interventions have been identified as resource-intensive and context-specific, limiting their applicability across diverse healthcare settings [19]. Given the unique cultural, ethnic, linguistic and health system context within which South Asian populations access CVD care, a region-specific synthesis is essential to understand what types of interventions have been tested for South Asian populations with CVD, how they have been delivered, and what evidence exists regarding their feasibility and reported effectiveness. A previous review by Ens *et al*., (2014) [5], examined medication adherence among patients with CVD in South Asia (including India, Pakistan, Sri Lanka) and amongst South Asian ethnic minorities in high-income countries (Norway, Denmark and Canada), providing valuable insights into prevalence, determinants and non-interventional aspects influencing adherence. However, that review did not specifically focus on interventional studies and covered literature up to 2015. Since then, a growing number of intervention-based studies have emerged across South Asian settings, reflecting an evolving research landscape and increased policy attention toward improving adherence and continuity of CVD care.

This systematic review provides a narrative synthesis of interventional studies aimed at improving medication adherence amongst people from South Asian ethnic groups with diagnosed CVD. This review seeks to (i) map the range of intervention types and delivery modalities evaluated, (ii) synthesise evidence on the reported effectiveness and feasibility of such interventions and (iii) identify gaps within the evidence to inform future design of culturally appropriate, person-centred medication adherence interventions for South Asian populations worldwide.

## Methods

### Study design

This systematic review was conducted to synthesise evidence from studies employing diverse methodologies, including randomised controlled trials (RCTs), quasi-experimental, observational and mixed-methods design, pertaining to interventions aimed at improving medication adherence among South Asian populations with cardiovascular diseases. The review followed the Preferred Reporting Items for Systematic Reviews and Meta Analyses (PRISMA) [20] guidelines to ensure methodological rigour and transparency (S1 File).

A narrative thematic synthesis, describing studies, was undertaken to integrate these findings across heterogeneous study designs enabling authors to identify patterns and relationships across the reported intervention strategies, adherence outcomes and contextual factors. This systematic review was not prospectively registered with PROSPERO.

### Study selection and search strategy

**Fig 1:**
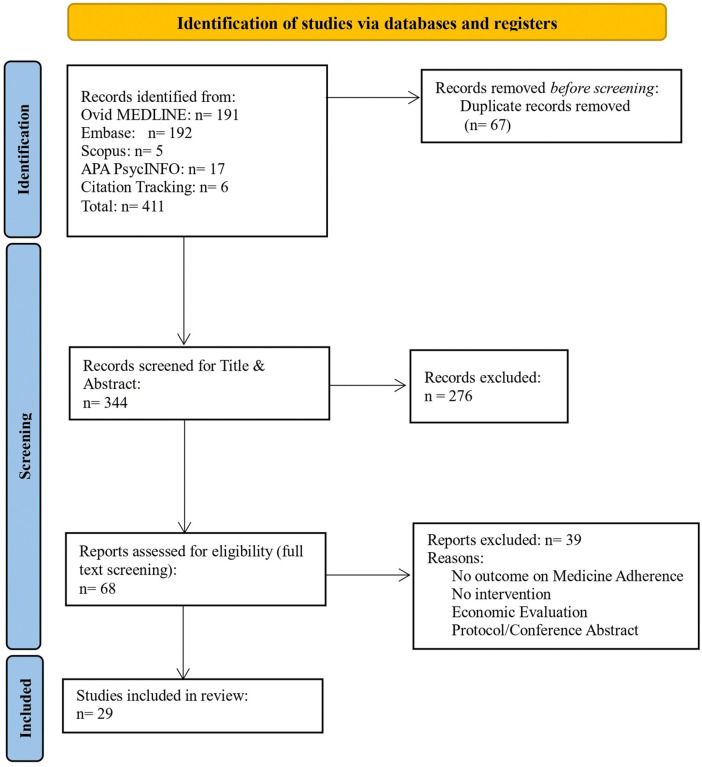
PICO framework used in the study. Icons sourced from Openclipart (https://openclipart.org/).

The study selection process was guided by the PICO framework (Fig 1), which defined the parameters for eligibility for inclusion of studies.

Based on this, the inclusion and exclusion criteria developed for screening the studies are outlined in Table 1.

**Table 1:** Inclusion and Exclusion Criteria.

Category	Inclusion Criteria	Exclusion Criteria
**Study Design**	Randomised controlled trials (RCTs), cluster randomised controlled trials, quasi-experimental studies, controlled before-and-after studies, cohort studies (prospective or retrospective), cross-sectional studies with an intervention component, qualitative and mixed-method studies, that reported relevant intervention outcomes.	Case reports, case series, editorials, letters to the editor, commentaries, protocols without outcome data, and conference abstracts without full-text availability and review articles (systematic, narrative, or meta-analyses).
**Population**	Adults aged 18 years and above; individuals of South Asian ethnicity (residing in South Asia or diaspora communities); participants diagnosed with cardiovascular diseases, including hypertension, coronary artery disease (CAD), coronary heart disease (CHD), myocardial infarction (MI), acute coronary syndrome (ACS), Ischaemic Stroke (IS), heart failure (HF), cardiac arrhythmias (*e.g.*, atrial fibrillation), stroke/cerebrovascular disease, or peripheral arterial disease (PAD), alone or in combination with diabetes.	Studies involving non-South Asian populations, paediatric populations (<18 years), or those without a clearly defined cardiovascular condition.
**Intervention & Outcome**	Studies that evaluated an intervention explicitly designed to improve medication adherence among individuals with CVD and reported it as an outcome.	Studies with no intervention component, interventions unrelated to medication adherence (e.g., diet-only or exercise-only interventions) or no outcome related to medication adherence.
**Publication Criteria**	Peer-reviewed full-text articles published in English between 2004 and 2025.	Grey literature, theses, conference abstracts, or non-peer-reviewed publications.

A comprehensive search was conducted using the electronic databases Ovid MEDLINE, Embase, Scopus, and APA PsycINFO, supplemented by citation tracking to identify additional relevant studies. The databases were searched from inception to 7 July 2025, which represents the date of the final search. The search strategy employed a combination of controlled vocabulary, MeSH terms and equivalent thesaurus terms specific to each database. Search terms were carefully refined using Boolean operators and adapted for each platform to ensure maximal retrieval of relevant records. To enhance sensitivity, key search terms also included the languages commonly spoken in South Asian countries (*e.g.,* Hindi, Urdu, Bengali, Tamil), which improved the identification of region-specific studies complementing the English-language MeSH terms. The search strategy employed the term ***‘South Asian diaspora’*** to encompass all the people of South Asian ethnicity living outside South Asia, regardless of migration status or generational distance from migration. This inclusive terminology was chosen in recognition that cultural factors influencing medication adherence may persist across generations and are not limited to the first-generation migrants alone. The search was limited to peer-reviewed journal articles published in English with full-text availability. A detailed description of the full search strategy and search history is provided in the Supplementary file (S2 File).

### Data extraction and management

All records retrieved from the database search were imported into Rayyan, a web-based tool used to streamline systematic review screening. Duplicate records were automatically identified and manually verified prior to removal. Title and abstract screening, followed by full-text assessment, were conducted independently by three reviewers (HMH, AH and AR-B) using Rayyan. Discrepancies were resolved through discussion with final decisions confirmed by one reviewer (HMH) to ensure consistency and transparency in study selection.

Data extraction was performed by one author (HMH) using Rayyan and subsequently exported to a Microsoft Excel for detailed analysis. The extracted data were independently checked by a second author (AR-B) to ensure accuracy and completeness. The extraction framework was adapted from the Joanna Briggs Institute (JBI) Data Extraction Template [21] and designed to systematically capture key study information, including: study identification details (author, publication year, country, and setting); study characteristics (design, sample size, and participant demographics such as age, sex and diagnosis); intervention details (type, delivery mode, provider, duration, and adherence measurement tools); and outcomes related to medication adherence and cardiovascular risk management.

### Data analysis

Data were analysed using a narrative synthesis approach, guided by Joanna Briggs Institute (JBI) methodology for systematic reviews. Extracted information on study characteristics, intervention type, delivery mode, duration and reported adherence outcomes for each study was collated. The compiled data were narratively synthesised to enable comparison across diverse study designs, populations and adherence measures. Key patterns and differences across interventions were identified and findings were iteratively organised into thematic areas reflecting intervention strategies, delivery approaches, contextual factors and adherence measurement.

### Quality assessment

The methodological quality of the included studies was appraised using the Joanna Briggs Institute (JBI) Critical Appraisal Checklists, selected for their applicability across diverse quantitative and mixed-method study designs. Specifically, the JBI critical appraisal checklist for RCTs, the checklist for Quasi-Experimental studies, the checklist for analytical cross-sectional studies were applied as appropriate to each study design. For mixed-methods studies, the relevant quantitative checklist was used in combination with the qualitative checklist. This approach provided a consistent and transparent framework for evaluating studies of varying methodological rigour, including RCTs, quasi-experimental designs, observational studies and mixed-method research. The summary of quality appraisal outcomes was tabulated, and studies were not excluded based on quality alone; rather, appraisal findings were used to inform the interpretation of evidence strength and the confidence in the overall synthesis.

### Certainty of evidence assessment (GRADE)

The certainty of evidence for key outcomes was assessed using the Grading of Recommendations Assessment, Development and Evaluation (GRADE) approach. Three critical outcomes were evaluated: (i) medication adherence, (ii) blood pressure control and (iii) sustainability of adherence over time. Evidence was initially rated according to study design and subsequently downgraded based on risk of bias, inconsistency, indirectness, imprecision and publication bias. A Summary of Findings table was generated to present overall certainty ratings.

## Results

### Study selection

**Fig 2:**
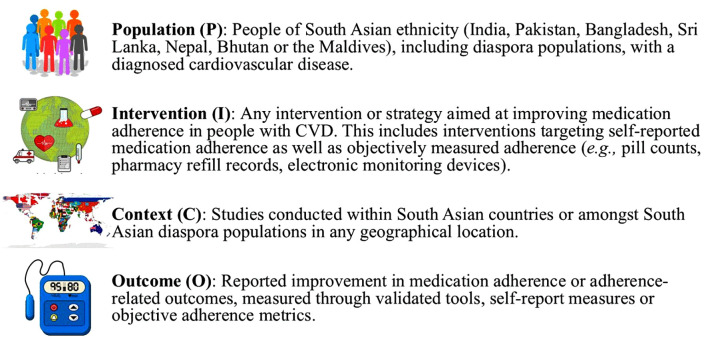
PRISMA flow diagram.

The database search yielded 411 records from Ovid MEDLINE, Embase, APA PsycINFO and Scopus, supplemented by forward and backward citation tracking. After the removal of 67 duplicates, 344 unique records were screened. Titles and abstracts were reviewed to exclude studies that did not meet the inclusion criteria, including those involving a different study population or reported as conference abstracts or case reports. Full texts of the remaining studies (n = 68) were then assessed in detail. At this stage, studies were excluded primarily because they did not include an intervention targeting medication adherence or they failed to measure medication adherence as an outcome. In total, 29 studies met the inclusion criteria and were included in the final synthesis, providing data on interventions designed to improve medication adherence among South Asian populations with cardiovascular diseases. The study selection process is presented in Fig 2.

### Study characteristics

The 29 included studies encompassed research conducted across South Asian countries including India [22–36], Pakistan [37–42], Nepal [43–47], Sri Lanka [39,48] and Bangladesh [39,49], with one study [50] examining South Asian ethnic minority populations residing in the United Kingdom.

The 29 included studies employed diverse methodological designs. Most were randomised controlled trials (RCTs) (n = 17), comprising traditional parallel-group RCTs (n = 11) [22,28,30,32,38,40–44,49], cluster RCTs (n = 3) [29,31,37] and feasibility/pilot RCTs (n = 3). [27,36,48] Quasi-experimental studies and interventional pilot programs constituted the second largest category (n = 8). These included pre-post designs (n = 5) [25,26,33–35], non-randomised comparison studies (n = 1) [47] and interventional feasibility pilots (n = 2).[23,50] The remaining research consisted of observational and descriptive designs (n = 4), including longitudinal or sustainability follow-up studies (n = 2) [24,46], a multi-country mixed-methods feasibility study (n = 1) [39] and one cross-sectional observational study (n = 1) [45].

Most interventions were implemented in tertiary hospital or outpatient clinical settings [22,24,25,29,30,32–36,38,40,41,43–45,47–49], although some were community-based, reflecting efforts to reach rural and under-resourced populations.[23,26–29,31,37,39,42,46,50]

Sample sizes varied considerably, ranging from small pilot trials with fewer than 50 participants [25,41,50] to large-scale community or multicentre studies involving more than 2000 individuals [32]. Hypertension was the most frequently studied condition, either as a primary focus or as a comorbidity within broader CVD cohorts [24–26,30,32,33,35–39,41–43,46,48]. Other studies included participants with acute coronary syndrome [28,29], myocardial infarction (MI) [34], stroke [40,45,47,49], coronary artery disease [40,50] or mixed CVD risk profiles. [22,23,27,31,44]

Participant mean ages ranged from the early 30–75 years, reflecting predominantly middle-aged and older adults at increased cardiovascular risk. Most studies included more male participants, though some community-based trials had balanced or female-majority samples. Collectively, the studies represented diversity in research methodologies used, as well as people from South Asian countries (Table 2).

**Table 2:** Study Characteristics of the 29 included studies.

Study/Author (year)	Specific place (country) within South Asia	Study Design and setting	Patient Population	Mean Age/Age distribution	Sex (as reported by the original authors of the study)
Ponnusankar *et al*. (2004) [22]	India (Ooty)	Randomised controlled trial in an outpatient clinic	90 Hypertensive and Diabetes Mellitus patients	45.4 (Counselled), 51.5 (Control)	63.33% Counselled group,31 (51.66%) Usual care group men; 11 (36.66%) Counselled group, 29 (48.33%) Usual care group women
Qureshi *et al.* (2007) [37]	Pakistan (Karachi)	Cluster Randomised controlled trial in 6 communities	200 Hypertensive patients; 78 GPs	55.3 years	75 (37.5%) men and 125 (62.5%) women
Kar *et al*. (2008) [23]	India (North)	Quasi-Experimental study in 8 health primary cares located in rural, urban, and slum areas of northern India	1410 CVD risk (Hypertensive) participants	30-75 years	415 (41.1%) men and 595 (58.9%) women
Bahl *et al*. (2009) [24]	India (Multiple centers)	Prospective, observational, multicenter trial (STRONG study) in outpatient clinic	1250 Hypertensive patients & 336 GPs	55.6 years	759 (60.7%) men, 491 (39.3%) women
Palanisamy *et al*. (2009) [25]	India (Coimbatore)	Cross-sectional descriptive comparative study in a tertiary care teaching hospital	43 Hypertensive patients	59.63 years	25 men, 18 women
Soliman *et al.* (2011) [48]	Sri Lanka	Open-label, parallel-group, Randomised clinical trial at 3 tertiary care hospitals	216 Hypertensive patients	59.1 years	157 (72.7%) women and 59 (27.3%) men
Sathvik *et al.* (2012) [26]	India (Rural South)	Quasi-Experimental study conducted in 3 rural areas	150 patients (75 each control group & intervention group)	51-60 years	79 women and 71 men
Saleem *et al.* (2013) [38]	Pakistan	Non-clinical Randomised controlled trial in cardiac units of Public Hospitals	385 Hypertensive patients	39 years	265 (68.8%) men, 120 (31.2%) women
Thom *et al.* (2013) [27]	India (and Europe)	UMPIRE trial: Randomised, open-label, blinded-endpoint trial in 28 hospital specialist clinics	1000 CVD South Asian patients	62.1 years	81.9% men, 18.1% women
Jafar *et al.* (2016) [39]	Bangladesh, Pakistan, Sri Lanka	Mixed-method feasibility study; pre- and post-evaluation in rural communities	412 Hypertensive patients were recruited out of 454 eligible individuals	59.8 years	122 (29.6%) men and 290 (70.4%) women
Sharma *et al.* (2016) [28]	India (Jaipur)	Randomised controlled trial in tertiary-care cardiac centre	100 Acute Coronary Syndrome (ACS) patients	59 years	84% men, 16% women
Xavier *et al.* (2016) [29]	India (Multiple centers)	Multicenter (hospitals), open, Randomised controlled trial	806 Acute Coronary Syndrome participants. 750 assessed at 1 year	56.4 years	83% men 17% women
Kamal *et al.* (2018) [40]	Pakistan	Randomised clinical trial (The Talking Rx Study) in Tertiary care outpatient setting	197 coronary artery disease (CAD) and Stroke participants	59.1 years	77% men, 23% women
Jalal *et al.* (2018) [50]	London, UK (South Asian community)	Feasibility pilot-controlled trial in London Heart Attack Centre and community pharmacy	17 South Asian patients (71 total, 54 non-South Asian) with coronary heart disease (CHD)	32-72 years (14 patients interviewed)	13 men, 1 woman (South Asian subgroup)
Amer *et al.* (2018) [41]	Pakistan	Randomised controlled trial in Hospital cardiology Outpatient Department (OPD)	384 Hypertensive patients (192 intervention, 192 control)	50.21 years	215(56%) men, 169 (44%) women
Sheilini *et al.* (2019) [30]	India (Karnataka)	Randomised controlled trial; experimental design in tertiary care hospital	160 Hypertensive patients (80 intervention, 80 control)	68.2 years	47.6% (59) males,52.4% (65) women
Joshi *et al.* (2019) [31]	India (3 states-Rural)	Open-label cluster-Randomised trial in 28 villages	2312 households with 3261 participants at intermediate/high CVD risk	61.7 years	2398 men (57.9%) and 1740 women (42.1%)
Shukla *et al.* (2020) [32]	India (New Delhi)	Prospective study in a tertiary health-care center	250 Hypertensive patients	57.7 years	men (75.6%), women (24.4%)
Kavitha *et al.* (2020) [33]	India (North)	Quasi-experimental study with 1-year follow-up in tertiary health care center	402 patients for primary prevention and 500 patients for secondary prevention of Hypertension	50-69 years	men (75.6%), women (24.4%)
Sundararajan *et al.* (2020) [34]	India (South)	Prospective interventional study in Tertiary care hospital	160 Post-Myocardial Infarction (MI) patients	56.38 years in Group A and 53.93 years in Group B	76% men in Group A; 86.67% in Group B, & 24% women in Group A; 13.33% in Group B
Bhandari *et al.* (2022) [43]	Nepal (Kathmandu)	Feasibility Randomised controlled trial (TEXT4 BP) in tertiary referral hospital	200 Hypertensive patients (100 intervention, 100 control)	50.5 years	44.5% women, 55.5% men
Andrew *et al.* (2022) [35]	India (Hyderabad)	Quasi-experimental study with a pretest-post-test control group design in Health centres	256 Hypertensive patients	52.2 years	53.3% men and 46.7% women
Paudel *et al.* (2022) [44]	Nepal	Randomised controlled trial in tertiary referral hospital	56 participants with Hypertension and/or T2DM	40 years	57% women, 43% men
Sarraf *et al*. (2023) [45]	Nepal (East)	Observational study in an outpatient department	105 patients with Hypertension & Stroke survivors	51.1 years	60.95% women, 39.05% men
Neupane *et al.* (2023) [46]	Nepal (Kaski)	COBIN open-label cluster Randomised trial in community	1638 Normotensive, prehypertensive, or hypertensive patients	45.3 years	964 (71.3%) women, 388 (28.7%) men
Babu *et al.* (2024) [36]	India (South)	Randomised controlled trial (MaMoRS) in tertiary care hospital setting	209 stroke survivors & Hypertensive patients	60.4 years	77% male, 23% women
Arshed *et al.* (2024) [42]	Pakistan (Lahore)	Randomised controlled trial (Multi-Aid-Package) in public tertiary care hospital	440 Newly diagnosed hypertension patients (423-completed study)	50 years	38.95% women, 61.05% men
Shrestha *et al.* (2024) [47]	Nepal	Quasi-experimental study in medical outpatient department	100 Stroke patients (78 completed study)	52.7 years	47% women, 53% men
Afrin *et al.* (2025) [49]	Bangladesh (Dhaka)	Randomised controlled trial at tertiary care hospital	432 Stroke Patients	55.2 years	63.2% men, 36.8% women

### Demographics

**Fig 3:**
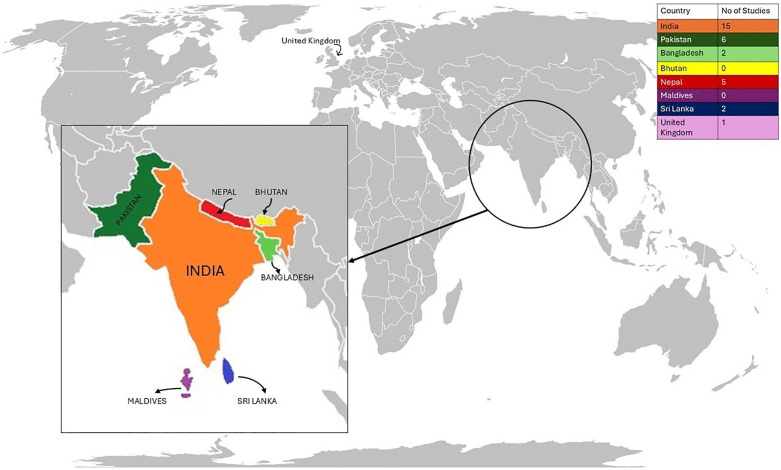
Geographic distribution of included interventional studies across South Asia. Base layer map: Equal Earth (public domain). Available at: https://equal-earth.com/.

The geographic distribution of the included studies demonstrated representation from five South Asian countries (Fig 3). India (n = 15) [22–36], Pakistan (n = 6; including one multi-country trial) [37–42], Nepal (n = 5) [43–47], Bangladesh (n = 2; including one multi-country trial) [39,49] and Sri Lanka (n = 2); including one multi-country trial) [39,48]. In addition, one study was conducted with people from South Asian ethnicity (specifically India, Pakistan, Bangladesh) who were residing in the United Kingdom. [50] No interventional studies meeting the inclusion criteria were identified from Bhutan or the Maldives.

### Intervention characteristics

Across the 29 included studies, there was heterogeneity in the style and type of interventions used, with the aim of supporting improvements in medication adherence and cardiovascular risk management (Table 3). These have been categorised into six types; (i) educational interventions were most common and encompassed strategies to educate patients or healthcare professionals [22,26,34,35,37–39,41,47,49] and used printed materials or provided structured training around adherence and lifestyle modifications; (ii) counselling and behavioural interventions delivered personalised advice on risk factors, lifestyle change and adherence barriers, often provided by nurses, community health workers, or non-physician health workers [23,28,29,31,33,39,46,47]; (iii) medication-based interventions simplified treatment using fixed-dose combinations or polypills to reduce burden [24,27,48]; (iv) pharmacist-led interventions integrated education and counselling [25,26,34,38,44,50]; (v) technology-enhanced (mHealth) interventions used text messaging, smartphone applications, or interactive voice systems to deliver reminders and tailored information [32,36,40,42,43,45]; and (vi) multicomponent interventions combined educational, counselling, medication management and digital strategies, often within community-based or multidisciplinary frameworks.[23,30,39,40,42]

**Table 3:** Intervention Characteristics.

Study/Author (year)	Intervention Type	Delivery Mode	Intervention Provider	Follow-up Duration	Adherence Measure Used	Original author statement on effectiveness
Ponnusankar *et al*. (2004) [22]	Educational (Counselling)	In-person (OPD)	Pharmacist	6 weeks	Pill count; Self-report	Effective. Significant improvement in adherence (*p* < 0.05); intervention group showed higher compliance than control.
Qureshi *et al.* (2007) [37]	Professional Education	Intensive GP training	GPs	5 months	Electronic Medication Event Monitoring System (MEMS) bottle, which recorded the exact time and date of each cap opening	Effective. Significant increase: 48.1% (Special Care) vs 32.4% (Usual Care) of doses taken (*p* = 0.048).
Kar *et al*. (2008) [23]	Counselling + Education	Home visits	NPHW	60 days	Self-reporting	Effective. Adherence improved significantly (*p* < 0.001); regular intake of anti-hypertensive medication (58.3% vs 34.8%) in the intervention area compared to the control area
Bahl *et al*. (2009) [24]	Medication (Fixed Dose Combination)	Clinical Practice	GPs/PCPs	3 months	Self-reporting	Effective. High adherence achieved in 94% of the FDC group; mean BP was reduced after 60 days (*p* < 0.0001 compared to baseline).
Palanisamy *et al*. (2009) [25]	Pharmacist Intervention and included counselling on drugs and lifestyle modifications, medication schedule reminders, and frequent telephone reminders from the pharmacist	Face-to-face + Phone	Pharmacist	9 months	Morisky Scale	Effective. Adherence increased from 0% at baseline to 95.4% at final follow-up following pharmacist intervention (*p* < 0.05)
Soliman *et al.* (2011) [48]	Medication (Polypill)	Monthly clinic visits	Specialists	15 months	Pill count; Self-report	Over 80% of patients randomised to the Polypill demonstrated >80% adherence, with only 19% reporting adherence below 80%. However, the study could not properly assess intervention effectiveness because the control group received similar individual drugs (82.9% vs 43.8% received antihypertensive drugs, *p* < 0.01), defeating the study’s objective to observe the Polypill’s treatment effect
Sathvik *et al.* (2012) [26]	Pharmacist Education	Home visits	Pharmacist	6 months	Beliefs about Medicines Questionnaire (BMQ)	Effective. Significant reductions in self-reported non-adherence within the intervention group across regimen (*p* = 0.04), belief (*p* = 0.04), recall (*p* = 0.01), and access (*p* = 0.04) BMQ domains at 60-day follow-up, with no significant changes in the control group
Saleem *et al.* (2013) [38]	Pharmaceutical Care intervention-that included patient education, a pocket-sized educational book on hypertension, information leaflets, and medication adherence cards	Hospital interview	Pharmacist	24 months	Drug Attitude Inventory (DAI-10) by a self-administered questionnaire	Effective. Mean medication adherence scores increased in the intervention group from –1.8 at baseline to 3.2 post-intervention, while no meaningful change was observed in the control group; the between-group difference was statistically significant (*p* < 0.001)
Thom *et al.* (2013) [27]	Medication (FDC)	Clinical Prescription	Physicians	60 days	Self-reported use	Effective. Adherence was 86% (FDC) vs 58% (Usual Care) (RR: 1.33; 95% CI, 1.26–1.41; *p* < 0.001)
Jafar *et al.* (2016) [39]	Multicomponent (MCI)	Home + Clinic visits	CHWs/GPs	3 months	Self-reported	Effective. The mean systolic blood pressure declined significantly by 4.5 mmHg (95% CI, 2.3–6.7; *p* < 0.001) in the overall pooled analysis of three countries
Sharma *et al.* (2016) [28]	Non-physician health worker (NPHW) led educational/behavioural adherence program targeting use of antiplatelets, β-blockers, RAS blockers, and statins, plus healthy lifestyles; personalised barrier assessment; use of visual calendar and health diary	Hospital/Home/Phone	Non-physician health workers (NPHW)	60 months	Composite Medication Adherence Score (CMAS); Visual Calendar	Effective. Adherence to various medicines in control and intervention groups at 12 and 24 months was > 80% (*p* < 0.001)
Xavier *et al.* (2016) [29]	Community Health Workers (CHWs)-led personalised adherence and lifestyle counselling using unstructured/relaxed discussions, barrier identification, the VITA visual calendar and a patient diary, plus monitoring of adherence and risk markers	Hospital/Home/Phone	Community Health Workers (CHWs)	3 months	Composite Medication Adherence Score (CMAS); Visual Calendar	Effective. Intervention significantly increased medicine adherence (97% in intervention group vs 92% in usual care group; OR 2.62, *p* = 0.006)
Kamal *et al.* (2018) [40]	Automated mHealth multicomponent intervention (“Talking Rx”): (a) Interactive Voice Response (IVR) tailored medication information (patient can call and hear their prescription “talk”), (b) daily tailored SMS medication reminders (statin + antiplatelet), and (c) once-weekly lifestyle-modification SMS. Plus, an investigator helpline.	Phone (IVR & SMS)	Automated SMS delivered 5 days/week	8 months	Morisky Medication Adherence Scale (MMAS-8)	Not effective. Mean MMAS-8 scores increased to 7.41 (SD 0.78) in the intervention group vs. 7.38 (SD 0.99) in the usual care group (Mean Difference: 0.03; 95% CI, -0.23 to 0.29; *p* = 0.40)
Jalal *et al.* (2018) [50]	Pharmacy-led (MUR/NMS)- Pharmacy care with motivational interviewing integrated into UK New Medicine Service (NMS) or Medication Use Review (MUR); plus, discharge counselling and referral to cardiac rehab at hospital discharge	Pharmacy Face-to-face	Pharmacist	6 months	Morisky Medication Adherence Scale (MMAS-8) (primary outcome). Beliefs about Medicines Questionnaire-Specific (BMQ-S) used to examine belief–adherence relationship.	Effective. At 6 months, mean adherence scores were 7.5 (93.75%) for the intervention group vs. 6.1 (76.25%) for the control group (*p* = 0.004)
Amer *et al.* (2018) [41]	Educational Program- printed booklet in Urdu-(hypertension-related information, lifestyle education, medication counselling tips to increase knowledge about hypertension, adherence to medication	Interview + Booklet	Pharmacist	6 months	Morisky Medication Adherence Scale-Urdu (MMAS-U)	Effective. Adherence score was 5.89 ± 1.90 (IG) vs 3.89 ± 1.19 (CG) (*p* < 0.001); SBP was 131.81 ± 10.98 mmHg (IG) vs 137.91 ± 12.02 mmHg (CG) (*p* < 0.001)
Sheilini *et al.* (2019) [30]	Multimodal Intervention-included individualised teaching on medication adherence and healthy lifestyle practice	Separate OPD Room	Nurse-led CHW	18 months	Morisky Medication Adherence Scale (MMAS-8); Pill count	Effective. Medication adherence mean score improved from 5.59 (baseline) to 8.00 (6 months) in the intervention group vs. 5.93 to 7.70 in the control group
Joshi *et al.* (2019) [31]	Risk-reduction/Advice: The advice included promoting adherence to prescribed medication, blood pressure measurements, and lifestyle advice on tobacco cessation, salt reduction, diet, and physical activity	Home visits	Community Health Workers (CHWs)	2 months	Morisky, Green, and Levine Adherence Scale, also known as the Medication Adherence Questionnaire (MAQ)	Effective. Adherence to antihypertensive drugs was 74.9% (Intervention) vs. 61.4% (Control) (*p* = 0.001); however, there was no significant difference in SBP between groups (*p* = 0.18)
Shukla *et al.* (2020) [32]	mHealth (SMS/WhatsApp): Mobile phone text messaging and social media-sending messages in the form of either SMS or WhatsApp regularly to remind participants about the importance of regular medicine intake	System Automated	Messages were sent regularly (once every 3 days)	1 year	Hindi version of the eight-item Morisky Medication Adherence Scale (MMAS)	Effective. High adherence increased from 34.8% at baseline to 88.4% at 2 months (*p* < 0.05); SBP decreased by 8.3 mmHg (*p* < 0.001) and DBP by 2.4 mmHg (*p* = 0.002)
Kavitha *et al.* (2020) [33]	Nurse-led Counselling	OPD + Telephone	Nurses	6 months	Medication Adherence Rating Scale (MARS)	Effective. Mean medication adherence score was 7.60 (Intervention) vs. 5.96 (Comparison) with a large effect size of 1.1 (*p* < 0.01)
Sundararajan *et al.* (2020) [34]	Clinical Pharmacist Education	Verbal counselling, patient information leaflets (PILs), and telephone follow-ups.	Clinical Pharmacist	3 months	Hill Bone Scale, (which has a sub-score specifically for medication adherence)	Effective. Medication adherence scores improved significantly from 2.91 ± 1.15 at baseline to 7.23 ± 0.77 at 6 months in the intervention group (*p* < 0.0001)
Bhandari *et al.* (2022) [43]	mHealth -Mobile phone text messaging intervention (TEXT4 BP)	SMS (3x weekly)	Automated	6 weeks	Hypertension Compliance Scale (HyCompS Scale)	Effective. Adherence score was 7.15 ± 1.13 (IG) vs 6.13 ± 1.34 (CG) (Mean Difference: 1.02; 95% CI, 0.67–1.36; *p* < 0.001); SBP was 133.00 ± 15.61 mmHg (IG) vs 138.80 ± 15.00 mmHg (CG) (*p* = 0.009)
Andrew *et al.* (2022) [35]	A knowledge intervention with two forms: Direct Interaction (DI) and Audio-Visual (AV). Each form was presented with two frequencies: single exposure and double exposure	Verbal/ Video	Doctor and potentially health psychologists and nursing professionals.	4 months	Morisky-Green-Levine (MGL) scale	Effective. Medication adherence scores significantly improved across all intervention groups (*p* < 0.001)
Paudel *et al.* (2022) [44]	Hospital pharmacist-delivered individualised pharmaceutical service (P-DIPS)	Face-to-face + Phone	Pharmacist	2 months	Eight-Item Morisky Medication Adherence Scale (MMAS-8)	Effective. Adherence (MMAS-8) score was 7.42 ± 0.69 (IG) vs 6.12 ± 1.13 (CG) (*p* < 0.001); SBP was 130.40 ± 8.86 mmHg (IG) vs 140.64 ± 11.23 mmHg (CG) (*p* < 0.001)
Sarraf *et al*. (2023) [45]	mHealth (SMS)	SMS (2x weekly)	Hospital	6 months	Morisky Medication Adherence Scale-4 (MMAS-4)	Effective. Adherence scores improved from 5.56 ± 1.15 at baseline to 6.35 ± 1.25 at 3 months (p < 0.001); SBP decreased from 144.38 ± 17.56 mmHg to 134.12 ± 13.56 mmHg (*p* < 0.001)
Neupane *et al.* (2023) [46]	Lifestyle Counselling; + blood-pressure measurement delivered by FCHVs counselling targeted physical activity, salt reduction, alcohol reduction, smoking cessation, stress reduction; FCHVs measured BP and assessed/referrals	Home visits	Female Community Health Volunteers (FCHVs)	1 year	Self-reporting	Partial. At 60-month follow-up, only 42.0% of hypertensive participants in the intervention group were taking antihypertensive medicines, compared with 49.2% in the usual-care group. Intervention did not improve long-term medication adherence and was 7.2 percentage points lower than usual care.
Babu *et al.* (2024) [36]	Smartphone-based personal monitoring app called MaMoRS (Medication Adherence and Management of Risk Factors for Secondary Prevention of Stroke	App (Videos/SMS)	Automated	6 months	Self-Efficacy for Appropriate Medication Adherence Scale (SEAMS) questionnaire and self-report	Effective. Adherence was 95.2% (Intervention) vs. 79.4% (Control) at 6 months (RR: 1.20; 95% CI, 1.07–1.34; *p* = 0.001); SBP was 125.7 ± 11.3 mmHg (IG) vs. 131.5 ± 14.3 mmHg (CG) (*p* = 0.002)
Arshed *et al.* (2024) [42]	mHealth (multi-aid): structured counselling program. The counselling was based on existing guidelines and included education, a video, and an opportunity for the patient to ask questions	WhatsApp (Live 24/7)	Doctor/IT	1 month	Medication Adherence Report Scale (MARS)	Effective. Adherence was 83.3% (IG) vs. 40.5% (CG) (*p* < 0.001); SBP was 128.27 ± 9.39 mmHg (IG) vs. 143.23 ± 13.92 mmHg (CG) (*p* < 0.001)
Shrestha *et al.* (2024) [47]	Health education program. The intervention included self-management education on monitoring blood pressure (BP), medication, diet, and exercise.	Video + In-person	Health care Provider	12 months	Self-reporting	Effective. Medication adherence scores (MMAS-8) significantly improved from 5.48 ± 1.25 to 6.35 ± 1.25 in the intervention group (*p* < 0.001)
Afrin *et al.* (2025) [49]	Health Education	Face-to-face + Booklet	Research Assistant Nurse/Physio	12 months	Self-reporting	Effective. While stroke recurrence did not significantly differ (*p* = 0.19), the intervention led to lower mortality rates (18.1% IG vs. 25.9% CG) and significant improvements in modifiable risk factor control, with systolic blood pressure reaching 125.1 ± 10.4 mmHg in the intervention group compared to 132.4 ± 12.8 mmHg in the control group (*p* < 0.01)

### Data synthesis

**Fig 4:**
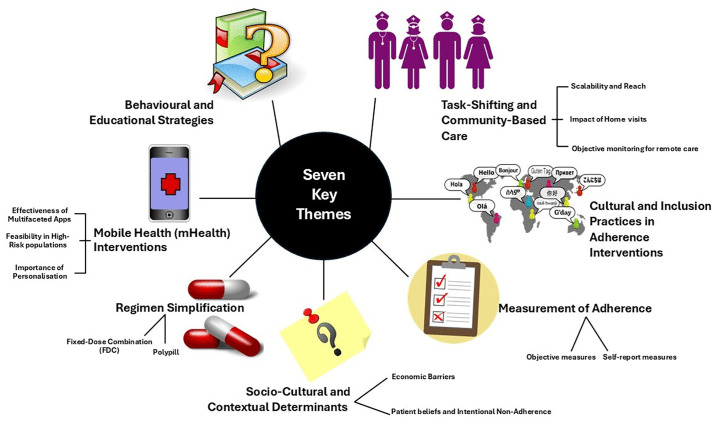
Seven key thematic areas in this review.

This synthesis integrated findings from the 29 included studies that examined interventions to improve medication adherence among patients from South Asian ethnic groups diagnosed with cardiovascular disease. Twenty-six studies reported statistically significant improvements in adherence (*p* < 0.05). The results are organised into seven key themes (Fig 4) and will be discussed in turn.

Icons sourced from Openclipart (https://openclipart.org/)

1. **Behavioural and Educational Strategies**

Interventions designed to have an impact on a person’s knowledge level or behaviour around medication adherence appeared successful, particularly when delivered through personalised, direct interactions with patients. The frequency and format of these interactions were key determinants of success. Specifically, knowledge-based interventions, when delivered by a healthcare professional and repeated at least twice, were found to improve both medication adherence and clinical parameters, such as blood pressure levels ((overall adherence M = 49.72–51.68 vs control M = 37.97, *p* < .001) and blood pressure control (mean arterial pressure M = 96.94–98.42 mmHg vs control M = 102.36 mmHg, *p* < .001)) [35]. Conversely, repeated exposure to recorded educational content was suggested to be potentially counterproductive, with the audio-visual double exposure group showing no significant difference from the control group in blood pressure management (mean arterial pressure: 100.28 mmHg vs control 102.36 mmHg, *p* = non-significant) [35].

Pharmacist-led interventions featured prominently and were notably effective; these involved pharmacists providing medication counselling, disease education and the use of culturally inclusive tailoring of educational materials through translation into local languages (*e.g.,* Urdu, Tamil, Hindi, Kannada, and Nepalese) (Table 4).

**Table 4:** Studies in which pharmacist-led education improved medication adherence.

Study	Key Finding
**Ponnusankar *et al.* (2004)** [22]	Pharmacist-led education significantly improved adherence (measured by pill count) from 59.4% (baseline) to 86.8% (post-intervention) (*p* < 0.05), and subsequently improved blood pressure control (37.2% to 76.7%; *p* < 0.05).
**Sathvik *et al.* (2012)** [26]	Pharmacist education led to a significant increase in the mean adherence score (MMAS-4) in the intervention group from 1.33 ± 0.72 to 3.49 ± 0.51 (*p* < 0.001).
**Sundararajan *et al.* (2020)** [34]	Found that pharmacist counselling combined with clinical care enhanced self-reported medication adherence (MARS score: intervention group M = 9.48, SD = 0.16 vs control M = 7.50, SD = 0.17, *p* < 0.0001) and lifestyle modifications post-myocardial infarction, with significant reductions in systolic blood pressure (131.72 mmHg to 122.80 mmHg, *p* = 0.0031), diastolic blood pressure (84.13 mmHg to 81.09 mmHg, *p* = 0.0069), and total cholesterol levels (224.70 mg/dL to 194.72 mg/dL, *p* = 0.0001).
**Amer *et al.* (2018)** [41]	Reported improved adherence following pharmacist-led education and follow-up visits in Pakistan, which included the provision of a printed educational booklet in the Urdu language.

Furthermore, targeted counselling, defined as a structured programme providing diagnosis-specific behavioural support, proved highly effective for specific patient groups. For example, Shrestha *et al.,* (2024) [47] reported success with a structured counselling programme for patients with newly diagnosed essential hypertension, focusing on specific elements such as knowledge of blood pressure targets, recommended salt intake, and required lifestyle changes; the intervention group demonstrated significantly higher post-counselling knowledge scores (mean = 6.6, SD = 1.29) compared to pre-counselling scores (mean = 4.5, SD = 1.21, *p* < 0.001) and superior medication adherence (median MARS score: 50 [IQR 48–50] *vs.* control 48 [IQR 43.5-50], p = 0.015). Whilst most studies reported both adherence and clinical improvements, some interventions were noted to improve adherence without producing significant clinical improvements; notably, Afrin *et al.,* (2025) [49] found that while a 12-month health education programme significantly improved medication adherence (84% taking medication 6–7 days/week in the intervention group *vs.* 71.6% in control, *p* = 0.002), it did not significantly reduce stroke recurrence (6.5% vs 3.7%, *p* = 0.19). This highlights the need for complex, sustained interventions that bridge the knowledge-to-outcome gap.

1. **Task-Shifting and Community-Based Care**

Task-shifting, an approach involving the rational redistribution of tasks among health workforce teams (*e.g.,* transferring tasks from physicians to non-physician personnel), emerged as a scalable and cost-effective model for non-communicable disease (NCD) management in resource-limited settings. In this model, non-physician personnel, primarily community health workers (CHWs) functioned as lay-professional workers who were often the closest link between the community and the formal health system. [51] This model allowed for community-based care to deliver services directly in patients homes or local settings, thereby tackling logistical barriers and increasing accessibility.

Kar *et al.,* (2008) [23] demonstrated that primary health workers in Northern India successfully implemented an adapted World Health Organization (WHO) CVD risk management package. Their role focused on risk screening and adherence reinforcement under physician oversight. This showed that task-shifting can efficiently extend scalability and reach of NCD care beyond centralised clinics.

The impact of home visits was explored by Joshi *et al.,* (2019) [31], where CHWs performed bi-monthly home visits for blood pressure monitoring and adherence reinforcement. The key impact of these community-based visits is their ability to address local, individual-level barriers (*e.g.,* poverty, access, time constraints) at the point of care. At the 12-month follow-up, adherence to antihypertensive therapy was significantly higher in the intervention group (74.9%; 95% CI:70.2-79.0%) compared to the control group (61.4%; 95% CI: 56.1-66.4%; *p* = 0.001). However, this effect diminished to 49.3% six months after the home visits ceased, suggesting that the benefits of community-based reinforcement may require sustained delivery to ensure long-term sustainability.

Qureshi *et al.,* (2007) [37] used electronic Medication Event Monitoring System (MEMS) bottles to objectively record the date and time of cap opening as a measure of adherence. By having CHWs download this objective adherence data, they were able to facilitate remote physician consultation as a form of data-driven treatment adjustments (*e.g.,* dose escalation).

1. **Mobile Health (mHealth) Interventions**

Digital interventions, typically delivered *via* mobile phones, provided scalable and low-cost support to positively influence medication adherence. These interventions included automated SMS/WhatsApp text message alerts, notifications and multifaceted health applications (apps). [32,36,40,42,43]

Multifaceted health applications (apps) demonstrated significant positive outcomes. For instance, Arshed *et al.*, (2024) [42] evaluated a comprehensive mHealth application (Multi-Aid Package) and reported a statistically significant improvement in adherence scores and concomitant reduction in systolic blood pressure (SBP) over six months. The median - Self-Efficacy for Appropriate Medication Adherence Scale (SEAMS) adherence score increased by 12.5 points in the intervention group (*p* < .001), with 37.7% of participants achieving adherent status, whilst median systolic blood pressure decreased by 4 mmHg (*p* < .001), and 22.3% achieved blood pressure control (<140 mmHg). Similar positive results were reported by Shukla *et al.,* (2020) [32] and Bhandari *et al.,* (2022) [43], indicating that digital reminders *via* messaging are effective in improving both adherence and blood pressure control.

Babu *et al.,* (2024) [36] tested an app intervention tailored for stroke survivors in India, concluding that the system was feasible (64% of participants used their own devices) and effective, with mean medication adherence scores improving significantly more in the intervention group (from 2.46 to 3.61) compared to control (from 2.44 to 2.88), yielding a between-group difference of 0.735 (95% CI 0.419 to 1.050, *p* < .001) for improving medication adherence and managing vascular risk factors in this high-risk population.

Crucially, not all digital interventions proved effective. An Interactive Voice Response (IVR)/SMS-based system, which lacked personalised engagement and interaction, showed limited impact on medication adherence.[40] The mean MMAS-8 scores increased in both groups at 3 months, with a larger improvement in the intervention group (+0.73) *vs*. usual care (+0.61); however, the between-group difference was not statistically significant (*p* = 0.40). While the system was found to be feasible and well-received by end-users, this finding suggests that the success of mHealth interventions depends less on the technology itself and more on the degree of personalised, sustained patient interaction or complexity of the intervention.

1. **Regimen Simplification**

Interventions that simplify complex medication schedules by combining multiple drugs into a single pill were associated with improved adherence.

Bahl *et al.,* (2009) [24] showed that fixed-dose combinations therapies, such as perindopril/amlodipine combination, improved blood pressure control in hypertensive patients. There was a mean reduction in SBP of 41.9–34.8mmHg at day 60 (to 125.4–33.1mmHg; *p* < 0.001 *vs*, baseline) representing a decrease by 25%. The underlying mechanism is the reduction of pill burden and simplification of the dosing schedule, which is a powerful technique for overcoming unintentional non-adherence (*e.g.,* forgetting multiple pills).

Large, multicentre trials demonstrated the effectiveness of the polypill-a single capsule containing a combination of antiplatelet, statin and antihypertensive agents. There was > 80% adherence to the pill [27,29,48] indicating that the polypill significantly improved medication adherence among patients requiring secondary prevention of CVD, confirming the clinical utility of regimen simplification in long-term disease management.

1. **Socio-Cultural and Contextual Determinants**

Medication cost was identified as a major barrier [25], especially when compounded by lower socioeconomic income and lower rates of health literacy [26]. Palanisamy *et al.,* (2009) [25] stated that unintentional non-adherence was most frequently reported as ‘Forget’ (72.09%), while ‘too expensive (18.60%) was also a significant barrier.

Patient beliefs were crucial drivers of intentional non-adherence. Palanisamy *et al.,* (2009) [25] found that the highest reason for intentional non-adherence was perceived adverse effects (74.42%), followed by patients doubting medication efficacy (39.53%). Conversely, Jalal *et al.,* (2018) [50] found that a strong belief in the necessity of medication was statistically correlated with better adherence. At 3 and 6 months, adherence was significantly higher in the intervention group compared with controls (3 months: 7.7 *vs.* 7.0, *p* = 0.026; 6 months: 7.5 *vs*. 6.1, *p* = 0.004). This improved adherence at 3 months was significantly associated with stronger necessity beliefs on the BMQ-S (*p* = 0.028).

Cultural and religious practices frequently contributed to non-adherence. Palanisamy *et al.,* (2009) [25] reported that religious fasting once per month accounted for 39.53% of intentional non-adherence reasons. Furthermore, qualitative interviews Jalal *et al.,* (2018) [50] highlighted that culturally specific beliefs, such as attributing illness to fate, genetics or ‘God’s will’, influence a patient’s perception of their control over prevention and their motivation for long-term adherence.

1. **Measurement of Adherence**

The assessment of medication adherence utilised a hybrid of objective and self-reported methods across the studies, reflecting the challenges of accurate adherence measurement. Objective measurement methods were used in some studies and included approaches such as MEMS bottles [37], pill counts, and calculations of consumed *vs.* prescribed doses [22].

The most frequently employed self-report instrument was the Morisky Medication Adherence Scale (MMAS), used in its 8-item (MMAS-8), 4-item (MMAS-4), and original MGL-item forms [30,32,33,45]. Other validated self-report tools included the Medication Adherence Report Scale (MARS) [34,47], the Drug Attitude Inventory (DAI-10) [38] and condition-specific instruments such as the Hypertension Compliance Scale (HyCompS) [35]. Additionally, the Beliefs about Medicine Questionnaire-Specific (BMQ-S) was used to assess the influence of patient cognition on adherence behaviour. [50]

1. **Cultural and Inclusion Practices in Adherence Interventions**

A dedicated focus on cultural and inclusion practices emerged as an essential component of successful adherence strategies, moving beyond simple medical management to address the patient’s lived context. The most common approach utilised inclusive language practices where pharmacist-led interventions tailored educational content by translating materials into local languages (Table 5).

**Table 5:** Studies involving inclusive language practice.

Study	Inclusive Language Practice
Ponnusankar *et al. (*2004) [22]	The self-assessment form for medication adherence was bilingual in English and Tamil (local language).
Sheilini *et al. (2019)* [30]	Patients were required to converse in English/Kannada. The patient information leaflet on adherence was prepared in both English and Kannada.
Kamal *et al. (2018)* [40]	Informed consent was available in both English and Urdu.
Amer *et al. (2018)* [41]	A printed booklet of hypertension-related educational material was provided in the Urdu (local) language.
Sathvik *et al. (2012)* [26]	Found a high proportion of patients were illiterate (n = 85), highlighting the importance of intervention format beyond text-based materials (verbal education in local language).

### Quality assessment

Thirteen studies were appraised as high quality [27–29,36,37,40,42–46,48,50], characterised by robust randomisation, secure allocation concealment and blinded outcome assessment. Most other randomised controlled trials (RCTs) were deemed moderate quality [22,30–32,38,41,49], frequently due to the unavoidable lack of participant blinding in behavioral or counselling interventions, which is a common challenge in non-pharmacological trials or the incomplete reporting of concealment methods.

Quasi-experimental studies were similarly classified as moderate quality [23,26,33,35,39,47], reflecting inherent selection bias from non-random allocation, though they maintained consistent outcome measurement. In contrast, single-arm pre–post studies without control groups were graded as low quality [25,34], as their findings remain vulnerable to confounding variables and maturation effects. The qualitative component of the feasibility evidence [50] was rated high quality, providing nuanced, culturally specific insights into the drivers of adherence. (Table 6)

**Table 6:** Quality Assessment of 29 included studies using JBI checklist.

Study (Author)	Study Design Identified	JBI Checklist Used	Overall Quality Rating	Rationale
Ponnusankar *et al.* (2004) [22]	RCT	JBI RCT	Moderate	Randomised 1:2. Lack of detail on allocation concealment and blinding of assessors.
Qureshi *et al.* (2007) [37]	Cluster RCT	JBI RCT	High	Cluster Randomisation at GP level. Used objective MEMS monitoring; high follow-up rate.
Kar *et al*. (2008) [23]	Quasi-Experimental	JBI Quasi-E.	Moderate	Non-Randomised comparison. High risk of selection bias due to purposive area selection.
Bahl *et al*. (2009) [24]	Observational Cohort	JBI Cohort	Moderate	Prospective single-arm study. No control group, but strong objective BP data.
Palanisamy *et al*. (2009) [25]	Quasi-Experimental	JBI Quasi-E.	Low	Single-arm pre-post design. No control group; significant risk of confounding.
Soliman *et al.* (2011) [48]	RCT (Feasibility)	JBI RCT	High	Clear randomisation and Intention-to-Treat (ITT) analysis. High completion rate (94%).
Sathvik *et al.* (2012) [26]	Quasi-Experimental	JBI Quasi-E.	Moderate	Used a control group but lacked detail on the equivalence of groups at baseline.
Saleem *et al.* (2013) [38]	RCT	JBI RCT	Moderate	Randomisation mentioned; however, blinding of participants/personnel was not possible.
Thom *et al.* (2013) [27]	RCT (Feasibility)	JBI RCT	High	Part of the UMPIRE trial; robust Randomisation and large sample size (2004).
Jafar *et al.* (2016) [39]	Mixed-Methods	JBI Quasi-E.	Moderate	Pre-post evaluation of a feasibility package across three countries.
Sharma *et al.* (2016) [28]	RCT	JBI RCT	High	Clear randomisation, 90% power, and long-term (24-month) follow-up reporting.
Xavier *et al.* (2016) [29]	Cluster RCT	JBI RCT	High	Robust cluster design; clear ITT analysis and baseline comparability.
Kamal *et al.* (2018) [40]	RCT	JBI RCT	High	Used computerised randomisation, allocation concealment, and blinded assessors.
Jalal *et al.* (2018) [50]	Interventional Pilot	JBI Quasi-E.	High	High-quality pilot design; effectively explored beliefs via qualitative sub-analysis.
Amer *et al.* (2018) [41]	RCT	JBI RCT	Moderate	Randomisation reported. Unblinded intervention; potential detection bias.
Sheilini *et al.* (2019) [30]	RCT	JBI RCT	Moderate	Randomised by lot-drawing. High risk of performance bias in educational setting.
Joshi *et al.* (2019) [31]	Cluster RCT	JBI RCT	Moderate	Cluster randomisation. Reliance on self-reported adherence (detection bias).
Shukla *et al.* (2020) [32]	RCT	JBI RCT	Moderate	Randomised to SMS/WhatsApp. Short duration (2 months); no assessor blinding.
Kavitha *et al.* (2020) [33]	Quasi-Experimental	JBI Quasi-E.	Moderate	Self-identified as quasi-experimental. 1-year follow-up; used nurses for delivery.
Sundararajan *et al.* (2020) [34]	Quasi-Experimental	JBI Quasi-E.	Low	Single-arm pre-post study. High risk of bias; reliance on self-report only.
Bhandari *et al.* (2022) [43]	RCT	JBI RCT	High	Robust randomisation and sound management of attrition.
Andrew *et al.* (2022) [35]	Quasi-Experimental	JBI Quasi-E.	Moderate	Non-equivalent group design.
Paudel *et al.* (2022) [44]	RCT	JBI RCT	High	Validated tool (MMAS-8) used. Clear randomisation and inclusion criteria.
Sarraf *et al*. (2023) [45]	Observational Cross-Sectional	JBI Cross-S.	High	Used validated MMAS-8; clear description of setting and participants.
Neupane *et al.* (2023) [46]	Cluster RCT	JBI RCT	High	Sustainability follow-up. Rigorous ITT and mixed-model statistical analysis.
Babu *et al.* (2024) [36]	RCT (Feasibility)	JBI RCT	High	Computer-generated randomisation; clear primary adherence outcomes.
Arshed *et al.* (2024) [42]	RCT	JBI RCT	High	Clear allocation concealment and blinded outcome assessment.
Shrestha *et al.* (2024) [47]	Quasi-Experimental	JBI Quasi-E.	Moderate	Non-randomised comparison. Used an adapted counselling program.
Afrin *et al.* (2025) [49]	RCT	JBI RCT	Moderate	Unblinded intervention; however, used blinded outcome assessment.

Despite the variation in quality, several cross-cutting methodological limitations restrict the comparability and generalisability of the evidence. First, there was a heavy reliance on self-reported adherence instruments (*e.g*., MMAS, MARS, or VAS), which are susceptible to overestimation due to recall and social desirability bias. Second, follow-up durations were often insufficient (frequently ≤ 6–12 months) to assess the long-term sustainability of adherence. This is particularly concerning as several studies observed a significant decline in adherence or clinical benefits once the active intervention ceased [31,33,46]. Furthermore, some trials were compromised by baseline imbalances or potential group contamination, which may have biased the reported effect sizes.

### Certainty of evidence (GRADE)

The overall certainty of evidence for improvement in medication adherence was rated as moderate. Although most included studies were RCTs demonstrating statistically significant improvements, the certainty was downgraded by one level due to concerns regarding risk of bias, particularly the reliance on self-reported adherence measures and limited blinding in behavioural interventions. The certainty of evidence for blood pressure control was rated as moderate. While multiple RCTs demonstrated significant reductions in systolic and diastolic blood pressure, heterogeneity in intervention type, duration and follow-up, alongside reliance on self-reported adherence measures, reduced confidence in the consistency of the observed effects. The certainty of evidence for sustained long-term adherence beyond the active intervention phase was rated as low. Although short-term improvements were frequently reported, evidence regarding long-term maintenance was limited, heterogeneous and often derived from studies with short follow-up durations. This reduces confidence in the durability of intervention effects. The summary of findings is presented in Table 7.

**Table 7:** Certainty of Evidence (GRADE).

Outcome	No. of studies	Study type	Overall effect	Certainty (GRADE)	Reason for Downgrading
Medication adherence	29	RCTs + quasi-experimental	26/29 improved adherence	**Moderate**	Risk of bias (self-report, lack of blinding)
Blood pressure control	~20	Mostly RCTs	Significant SBP/DBP reduction in most studies	**Moderate**	Risk of bias, heterogeneity
Sustainability of adherence	Limited subset	Mixed designs	Decline after intervention cessation in several studies	**Low**	Risk of bias, inconsistency, short follow-up

## Discussion

This systematic review synthesised evidence from 29 studies examining interventions designed to improve medication adherence and cardiovascular risk management among South Asian populations. An earlier review by Ens *et al.,* in 2014 [5], identified barriers to cardiac medication adherence amongst people from South Asian ethnic groups, including religious fasting, distrust of generic medicines, language barriers and family influence. This systematic review extends beyond these findings, by focusing exclusively on interventional studies conducted between 2004–2025; consequently, this work has shifted the discourse from identifying barriers to evaluating potential, evidence-based solutions. Specifically, this review has demonstrated the effectiveness and feasibility of several evidence-based strategies like polypills for pharmacological simplification, mHealth tools for behavioural reinforcement and task-shifting models (CHWs and pharmacists) for scalable service delivery. Crucially, this work has also identified the need for culturally tailored, faith-sensitive approaches to address intentional non-adherence, which represents a significant opportunity for researchers to explore in future trials.

The evidence synthesised here strongly supports the implementation of medication-based strategies, such as polypills (FDCs), for pharmacological simplification from wider literature. Consistent with the findings from major international trials, the process evaluation of the UMPIRE trial [52], which included sites in India, demonstrated that the simplicity of a once daily polypill significantly improved adherence compared to the complex regimens of usual care for CVD prevention. This simplifying effect was most pronounced in patients who were non-adherent at baseline, directly addressing a key challenge in long-term disease management within this population. Furthermore, linking to socioeconomic factors identified in the included studies, there remains an intersection where the reduction in the number of separate prescriptions could also mitigate the financial burden often associated with multiple medications, positioning the polypill as a cost-effective solution in resource-limited settings. However, translating this evidence into practice remains constrained by the limited real-world availability of FDC formulations within public health systems of many middle-income countries; the PURE study [53,54] found that availability of recommended CVD preventive medications fell below 60% across most LMIC communities, and as low as 3% in some settings. This availability gap represents a critical implementation barrier that warrants attention in future health policy and systems research.

Similarly, mHealth tools demonstrated high potential for scalable behavioural reinforcement, particularly in low- and middle-income South Asian settings. A formative qualitative study [55] conducted in Kathmandu, Nepal, confirmed that a simple text-messaging strategy was both acceptable and perceived as useful by patients and healthcare workers. This utility stemmed from the direct reinforcement of medication adherence, acting as a timely reminder, and strengthening recommended healthy behaviours like physical activity also supported by a study conducted on South Asians in New Zealand [56]. Text message approaches were deemed a possible cost effective and feasible mechanism for increasing patient coverage with minimal infrastructure investment. Critically, to achieve maximal acceptability and combat intentional non-adherence, the evidence highlighted the necessity for cultural and linguistic tailoring, including delivering messages in the local language. This necessitates a transition from linguistic translation to cultural competence (the ability of providers to recognise and respect the social and cultural values of the South Asian diaspora). Studies suggest that when interventions move from generic health advice to individualised care plans that account for a person’s specific health beliefs and literacy levels, patient trust and long-term engagement significantly increase [26,50]. Such individualisation ensures that the intervention is not just ‘available’ in their language, but ‘meaningful’ within their cultural framework.

The identified challenge of digital illiteracy [57] was also paired with a practical solution involving a family member to assist elderly or illiterate patients, providing a clear implementation pathway for addressing systemic barriers in a scalable manner. This multi-level approach to tackling systemic and implementation barriers, specifically leveraging family support and basic technology, educational outreach in resource-limited settings, is increasingly supported by the growing body of literature in the broader field of long-term disease management.[11,12,19,50,56]While the involvement of family members can provide a pragmatic solution to challenges with digital literacy, a tension exists between utilising familial support and fostering individual patient empowerment. Over-reliance on family may inadvertently create stigma or pressure, potentially undermining the patient’s autonomy. To address this, the evidence suggests that sustained professional reinforcement such as home visits by CHWs or nurses may be more effective than family-led support alone. As noted in the synthesis, the significant drop in adherence once home visits ceased [31,33,46] indicates that professional ‘human-in-the loop’ models provide a level of objective, structured accountability that family members cannot always maintain. Therefore, future implementation should prioritise nurse-led or CHW-led community outreach as a primary strategy, using family support only as a secondary, auxiliary tool.

The most persistent and underexplored limitation within the current evidence concerns the intersection of socio-cultural and contextual factors that shape an individual’s medication adherence behaviours. Effective adherence requires not only patient knowledge but also motivation and belief in self. Jalal *et al.,* (2018) [50] found that patients often attributed coronary artery disease to ‘God’s Will’, implying limited personal control and undermining sustained prevention efforts. Similarly, Palanisamy *et al.,* (2009) [25] reported that religious fasting contributed to intentional non-adherence. However, these findings highlight a critical disconnect; - although faith-based factors are central to adherence behaviour, no study to date has tested a structured faith-integrated intervention to support medication adherence for South Asian populations. Future research should seek to develop and evaluate culturally and religiously sensitive models that address fatalistic beliefs, incorporate faith leaders and provide practical guidance around medication adherence (for example, dose timing during fasting periods).

A primary strength of this systematic review is its comprehensive synthesis of diverse intervention modalities across multiple South Asian nations and the diaspora, offering a broad perspective on CVD medicine adherence strategies. However, a finding of note from this synthesis is the notable geographical imbalance across the existing evidence base; most studies were conducted in India, with few from Pakistan, Bangladesh, Sri Lanka and Nepal, which potentially limits regional generalisability for members of the South Asian community affected by CVD. Most studies focused on majority populations rather than minority or marginalised groups, leaving important gaps in understanding adherence behaviours across diverse communities. There was a lack of interventional studies from Bhutan or the Maldives, which likely reflects limited research infrastructure, small populations or publication barriers from these places, rather than an absence of a clinical problem (given that both countries have a significant burden of cardiovascular risk factors).[58–60] Expanding research to under-represented countries, populations and minority groups is essential to develop culturally appropriate, region-wide strategies. It is also important to contextualise these findings against the wider burden of diabetes across South Asian populations, which frequently co-occurs with the cardiovascular conditions addressed in this review and independently contributes to cardiovascular morbidity and mortality. Within the International Diabetes Federation (IDF) South-East Asia region, an estimated 107 million adults are living with diabetes, projected to rise to 185 million by 2050 [61], while Pakistan (classified separately within IDF Middle East and North African region) has the highest age-adjusted diabetes prevalence of any country worldwide and the fourth highest absolute number of adults living with diabetes globally [62]. Given this scale of comorbidity, future adherence interventions for South Asian populations with CVD may benefit from addressing diabetes management alongside cardiovascular risk factors, rather than treating these as separate clinical priorities.

## Conclusion

This systematic review provides a narrative synthesis of interventional studies aimed at improving medication adherence amongst people from South Asian ethnic groups with diagnosed CVD. The findings from 29 studies highlighted a clear sustainability gap, where short-term technological or educational bursts were associated with poorer maintenance of long-term adherence, without sustained human interaction or professional follow-up. While pharmacological strategies, mHealth reinforcement, and task-shifting models were demonstrated to be effective and feasible, there remains a paucity of culturally tailored, faith-sensitive interventions designed to address non-adherence driven by individualised, socio-cultural beliefs. Future research could, therefore, seek to explore mechanisms centered in culture and religion as a means of providing individualised, practical guidance to support medication adherence, with a focus on how beneficial effects can be sustained over longer periods. This shift toward longitudinal rather than cross-sectional approaches to adherence, alongside context-specific socio-cultural adaptation incorporating objective adherence measures and individualised care models that engage faith-based factors, may offer a more effective pathway to improving long-term medication-taking behaviours in this population.

## Supporting information

S1 File Prisma 2020 Checklist. Attribution. Author: The PRISMA Group (lead authors: Matthew J. Page, Joanne E. McKenzie, Patrick M. Bossuyt, Isabelle Boutron, Tammy C. Hoffmann, Cynthia D. Mulrow, et al.) Available at: https://doi.org/10.1136/bmj.n71. Source: PRISMA Statement website (https://www.prisma-statement.org/citing-prisma-2020). Licence: Creative Commons Attribution 4.0 International (CC BY 4.0). This PRISMA 2020 Checklist is distributed under the terms of the Creative Commons Attribution 4.0 International License, which permits unrestricted use, distribution, and reproduction in any medium, provided the original author and source are credited.
(DOCX)

S2 File Search Strategy. (DOCX)

S3 File Studies Assessed in Full-Text. (DOCX)

S4 File Data Extraction- Included Studies. (XLSX)
