# Supplementary material for: Medication adherence interventions for South Asian populations with cardiovascular disease: A systematic review and narrative synthesis
Source: PLOS Glob Public Health. 2026 Aug 12;6(8):e0006809. doi: 10.1371/journal.pgph.0006809 (PMC13465864; doi:10.1371/journal.pgph.0006809)
Supplement: Supplementary file 2 [file pgph.0006809.s002.docx]

***Medication adherence interventions for South Asian populations with cardiovascular disease: A systematic review and narrative synthesis***

## **S2 File: Search Strategy**

**Database- Embase**

exp Cardiovascular Diseases/ or exp Heart Diseases/ or exp Hypertension/ or exp Coronary Disease/ or exp Heart Failure/ or exp Arrhythmias, Cardiac/

(cardiovascular disease* or heart disease* or coronary artery disease* or coronary heart disease* or myocardial infarction* or heart attack* or heart failure* or cardiac failure* or hypertension* or high blood pressure* or arrhythmia* or atrial fibrillation* or angina* or stroke* or cerebrovascular*).mp.

(CVD or CHD or CAD or MI or HF or AF or HTN).mp.

exp Medication Adherence/ or exp Patient Compliance/ or exp Treatment Adherence/

(medication adherence* or medicine adherence* or drug adherence* or therapeutic adherence* or treatment adherence* or medication compliance* or drug compliance* or treatment compliance*).mp.

(medication concordance* or medicine concordance* or therapeutic concordance*).mp.

(medication taking* or medicine taking* or pill taking*).mp.

(medication persistence* or treatment persistence*).mp.

(south asian* or indian* or pakistani* or bengali* or bangladeshi* or sri lankan* or nepali* or bhutanese* or tamil* or punjabi* or gujarati* or hindi* or urdu* or sikh* or hindu*).mp.

(desi or subcontinental or indo-pak*).mp.

(minority* or ethnic minority* or racial minority* or immigrant* or migrant* or diaspora*).mp.

exp Medication Adherence/ or exp Patient Compliance/ or exp Treatment Adherence/

exp Asian Continental Ancestry Group/

(south asian* or indian* or pakistani* or bangladeshi* or sri lankan* or nepali* or bhutanese* or maldivian* or tamil* or punjabi* or gujarati* or hindi* or urdu* or bengali* or desi or indo-pak* or marathi* or nepal*).mp.

(immigrant* or migrant* or diaspora* or non-resident indian* or nri or british asian* or south asian immigrant*).mp.

**Database: APA PsycInfo**

(south asia* or india* or pakistan* or bangladesh* or sri lanka* or nepal* or bhutan* or maldiv*).mp.

(tamil* or punjabi* or gujarati* or telugu* or malayali* or marathi* or Urdu* or Bengali*).mp.

(desi or subcontinental or indo-pak* or "south asia*").mp.

(immigrant* or migrant* or diaspora* or "non-resident indian*" or nri or "british asian*").mp.

exp "Cardiovascular Disorders"/ or exp "Heart Disorders"/ or exp "Hypertension"/

exp "coronary heart disease"/ or exp "Myocardial Infarctions"/ or exp "Heart Failure"/ or exp "Cardiac Arrhythmias"/

(cardiovascular disease* or heart disease* or coronary artery disease* or coronary heart disease* or myocardial infarction* or heart attack* or acute coronary syndrome* or MI or heart failure* or cardiac failure* or congestive heart failure* or CHF or HF or hypertension* or high blood pressure* or elevated blood pressure* or HTN or arrhythmia* or atrial fibrillation* or cardiac arrhythmia* or AF or angina* or chest pain* or coronary artery disease* or CAD or ischemic heart disease*).mp. [mp=title, abstract, heading word, table of contents, key concepts, original title, tests & measures, mesh word]

(CVD or CHD or IHD).mp. [mp=title, abstract, heading word, table of contents, key concepts, original title, tests & measures, mesh word]

exp "Treatment Compliance"/ or exp "Medicine Adherence*"/ or drug adherence*.mp. or medication compliance*.mp. or medicine concordance*.mp. or "medication adherence scale*".mp. [mp=title, abstract, heading word, table of contents, key concepts, original title, tests & measures, mesh word]

exp "Intervention"/ or exp "Treatment"/ or exp "Therapy"/

(intervention* or program* or strategy* or approach*).mp.

(education* or counseling* or counselling* or coaching*).mp.

(behavior* change* or behaviour* change* or lifestyle* change*).mp.

**Database: Ovid MEDLINE(R)**

exp Cardiovascular Diseases/ or exp Heart Diseases/ or exp Hypertension/ or exp Coronary Disease/ or exp Heart Failure/ or exp Arrhythmias, Cardiac/

(cardiovascular disease* or heart disease* or coronary artery disease* or coronary heart disease* or myocardial infarction* or heart attack* or heart failure* or cardiac failure* or hypertension* or high blood pressure* or arrhythmia* or atrial fibrillation* or angina* or stroke* or cerebrovascular* or ischemic heart disease*).mp.

(CVD or CHD or CAD or MI or HF or AF or HTN or IHD).mp.

exp Medication Adherence/ or exp Patient Compliance/ or exp Treatment Adherence/

(medication adherence* or medicine adherence* or drug adherence* or therapeutic adherence* or treatment adherence* or medication compliance* or drug compliance* or treatment compliance*).mp.

(medication concordance* or medicine concordance* or therapeutic concordance*).mp.

(medication taking* or medicine taking* or pill taking*).mp.

(medication persistence* or treatment persistence*).mp.

(south asia* or india* or pakistan* or bangladesh* or sri lanka* or nepal* or bhutan* or maldiv* or southern Asia).mp.

(desi or subcontinental).mp.

exp Asian Continental Ancestry Group/

(south asian* or indian* or pakistani* or bangladeshi* or sri lankan* or nepali* or bhutanese* or maldivian* or tamil* or punjabi* or gujarati* or hindi* or urdu* or bengali* or desi or indo-pak* or marathi* or telugu or Ceylonese*).mp.

(immigrant* or migrant* or diaspora* or non-resident indian* or nri or british asian* or south asian immigrant*).mp.

(intervention* or program* or initiative* or strateg* or trial* or pilot project* or service* or campaign* or prevention program* or lifestyle intervention* or behavioral intervention* or treatment intervention*).mp.
