# Supplementary material for: Medication adherence interventions for South Asian populations with cardiovascular disease: A systematic review and narrative synthesis
Source: PLOS Glob Public Health. 2026 Aug 12;6(8):e0006809. doi: 10.1371/journal.pgph.0006809 (PMC13465864; doi:10.1371/journal.pgph.0006809)
Supplement: Supplementary file 3 [file pgph.0006809.s003.docx]

***Medication adherence interventions for South Asian populations with cardiovascular disease: A systematic review and narrative synthesis***

## **S3 File: Studies Assessed in Full-Text**

A total of 411 studies were retrieved from Ovid MEDLINE, Embase, APA PsycINFO and Scopus, supplemented by forward and backward citation tracking. After deduplication, 344 studies were screened at a title and abstract level, of which 276 studies were excluded. The full texts for sixty-eight studies were subsequently assessed for eligibility, based on inclusion criteria. The following table details the study information and reasons for their inclusion or exclusion.

| **Study number** | **Study title** | **Author, year** | **Outcome of inclusion/exclusion decision and reason** |
| --- | --- | --- | --- |
| 1. | Results of a comprehensive coronary heart disease prevention program in South India | Vinayagam *et al.,* 2015 | Excluded: Conference Abstract |
| 2. | Evaluating the Effectiveness of a Tailored Intervention on Medication Adherence among CABG Patients at a Tertiary Care Hospital in Delhi | Vaid H.K *et al.,* 2023 | Excluded: Unable to open |
| 3. | Effectiveness and Acceptability of Mobile Phone Text Messaging Intervention to Improve Blood Pressure Control (TEXT4BP) among Hypertensive Patients in Nepal | Bhattarai B.B *et al.,* 2023 | Excluded: Wrong outcome |
| 4. | Efficacy of *Nardostachys jatamansi* (D.Don) DC in essential hypertension: A randomized controlled study | M D A Bhat et al., 2020 | Excluded: Wrong intervention |
| 5. | Primary care & community health worker-based quality improvement program improves hypertension outcomes in urban indian slums | Chakrabarti T et al., 2019 | Excluded: Conference Abstract |
| 6. | Using Indian Health Service (IHS) counseling techniques in an  independent community pharmacy to improve adherence rates  among patients with diabetes, hypertension, or hyperlipidemia | N N Colvin et al., 2018 | Excluded: Wrong Population |
| 7. | Impact of clinical pharmacist intervention on hypertensive women and patient outcomes at a tertiary care hospital | Lavanya S et al., 2018 | Excluded: Conference Abstract; unable to open |
| 8. | Non-physician health workers for improving adherence to medications and healthy lifestyle following acute coronary syndrome: 24-month follow-up study | Sharma et al., 2016 | Included |
| 9. | Using a tailored health information technology driven intervention to improve health literacy and medication adherence in a pakistani population with vascular disease | Kamal A.K et al., 2016 | Excluded: Conference Abstract |
| 10. | Compliance to Hypertension Treatment in Residents of a Fishermen Colony in District Kollam, Kerala | A N Aslami et al., 2015 | Excluded: Wrong intervention |
| 11. | Self-reported medication adherence pattern of rural Indian patients with hypertension | Sathvik *et al., 2012* | Included |
| 12. | Pharmacist intervention in improving hypertension-related knowledge, treatment medication adherence and health-related quality of life: a non-clinical randomized controlled trial | Saleem *et al.,* 2013 | Included |
| 13. | A Low-Cost Educational Intervention for Rural Hypertension Control: Lessons for National Scale-Up | Mondal & Kapoor, 2025 | Excluded: Lacked quality threshold |
| 14. | Effectiveness of a Health Education Program to Reduce Recurrence of Stroke by Controlling Modifiable Risk Factors in a Specialized Hospital in Bangladesh: Randomized Controlled Trial | Afrin *et al., 2025* | Included |
| 15. | Effectiveness of Structured Counseling Program for Patients with Newly Diagnosed Essential Hypertension | Shrestha *et al.,* 2024 | Included |
| 16. | Improving medication adherence among persons with cardiovascular disease through m-health and community health worker-led interventions in Kerala; protocol for a type II effectiveness-implementation research-(SHRADDHA-ENDIRA) | Menon et al., 2024 | Excluded: Study Protocol |
| 17. | Effectiveness of a Multifaceted Mobile Health Intervention (Multi-Aid-Package) in Medication Adherence and Treatment Outcomes Among Patients With Hypertension in a Low- to Middle-Income Country: Randomized Controlled Trial | Arshed *et al.,* 2024 | Included |
| 18. | A randomized controlled trial of medication adherence and management of risk factors for secondary prevention of stroke (MaMoRS) using a smartphone-based application | Babu et al., 2024 | Included |
| 19. | Development and Effect of a Coordinated Community Health Worker Intervention Model for the Stroke Survivors in Kerala, South India | Sylaja et al., 2024 | Excluded: Full article unavailable |
| 20. | Effect of Weekly Text Messaging Reminders on Medication Adherence in Hypertension and Type 2 Diabetes Mellitus at a Tertiary Care Center in Eastern Nepal | Sarraf *et al*., 2023 | Included |
| 21. | Feasibility and acceptability study of risk reduction approach for stroke prevention in primary care in Western India | Dhikav et al., 2023 | Excluded: Wrong Outcome |
| 22. | The Effects of Clinical Pharmacist Education on Lifestyle Modifications of Postmyocardial Infarction Patients in South India: A Prospective Interventional Study | Sundararajan *et al.,* 2020 | Included |
| 23. | Impact of hospital pharmacist-delivered individualised pharmaceutical service intervention on clinical and patient-reported outcomes in patients with hypertension: a randomised controlled trial | Paudel *et al.,* 2022 | Included |
| 24. | Enhancing adherence and management in patients with hypertension: Impact of form and frequency of knowledge intervention | Andrew *et al.,* 2022 | Included |
| 25. | Effectiveness and Acceptability of a Mobile Phone Text Messaging Intervention to Improve Blood Pressure Control (TEXT4BP) among Patients with Hypertension in Nepal: A Feasibility Randomised Controlled Trial | Bhandari *et al.,* 2022 | Included |
| 26. | Acceptability of a mHealth strategy for hypertension management in a low-income and middle-income country setting: a formative qualitative study among patients and healthcare providers | Bhandari B et al., 2021 | Excluded: Wrong study design, outcome |
| 27. | Effectiveness of community-based health education and home support program to reduce blood pressure among patients with uncontrolled hypertension in Nepal: A cluster-randomized trial. | Khanal MK et al., 2021 | Excluded: Wrong Outcome |
| 28. | Barriers and facilitators for treatment and control of high blood pressure among hypertensive patients in Kathmandu, Nepal: a qualitative study informed by COM-B model of behavior change | Bhandari B et al., 2021 | Excluded: No intervention; duplicate |
| 29. | Swāsthya, an integrated chronic condition management programme for families of patients with hypertension and diabetes mellitus: a study protocol for a randomised controlled trial | M D Saju et al., 2021 | Excluded: Wrong outcome |
| 30. | Effects of a lifestyle-related risk factor modification intervention on lifestyle changes among patients with coronary artery disease in Nepal | Gaudel et al., 2020 | Excluded: Wrong Outcome |
| 31. | The South Asian Healthy Lifestyle Intervention (SAHELI) trial: Protocol for a mixed-methods, hybrid effectiveness implementation trial for reducing cardiovascular risk in South Asians in the United States | Kandula et al., 2020 | Excluded: Wrong study design: protocol |
| 32. | Effect of 5-Minute Movies Shown via a Mobile Phone App on Risk Factors and Mortality After Stroke in a Low- to Middle-Income Country: Randomized Controlled Trial for the Stroke Caregiver Dyad Education Intervention (Movies4Stroke) | Kamal et al., 2020 | Excluded: Wrong Outcome |
| 33. | Medication Adherence among Hypertensive Patients Attending a Tertiary Care Hospital in Nepal | Roka T et al., 2020 | Excluded: No intervention, paper unavailable |
| 34. | Task shifting of cardiovascular risk assessment and communication by nurses for primary and secondary prevention of cardiovascular diseases in a tertiary health care setting of Northern India | Kavitha *et al., 2022* | Included |
| 35. | Measurement of adherence to home-based exercises among community-dwelling stroke survivors in India | Mahmood et al., 2020 | Excluded: Wrong intervention |
| 36. | A prospective study to assess the medication adherence pattern among hypertensives and to evaluate the use of cellular phone text messaging as a tool to improve adherence to medications in a tertiary health-care center | Shukla et al., 2020 | Included |
| 37. | Effectiveness and cost-effectiveness of a Yoga-based Cardiac Rehabilitation (Yoga-CaRe) program following acute myocardial infarction: Study rationale and design of a multi-center randomized controlled trial | Chandrasekaran et al., 2019 | Excluded: Wrong outcome; Protocol paper |
| 38. | Effect of a community-based intervention for cardiovascular risk factor control on stroke mortality in rural Gadchiroli, India: study protocol for a cluster randomised controlled trial | Kalkonde et al., 2019 | Excluded: Study Protocol |
| 39. | Cardiovascular risk factor reduction by community health workers in rural India: A cluster randomized trial | Joshi *et al.,* 2019 | Included |
| 40. | Technology enabled non-physician health workers extending telemedicine to rural homes to control hypertension and diabetes (TETRA): A pre-post demonstration project in Telangana, India | Dandge et al., 2019 | Excluded: Wrong outcome |
| 41. | Statistical analysis plan for the control of blood pressure and risk attenuation-rural Bangladesh, Pakistan, Sri Lanka (COBRA-BPS) trial: a cluster randomized trial for a multicomponent intervention versus usual care in hypertensive patients | Gandhi et al., 2018 | Excluded: Protocol |
| 42. | Impact of pharmacist's intervention on disease related knowledge, medication adherence, HRQoL and control of blood pressure among hypertensive patients | Amer *et al.,* 2018 | Included |
| 43. | Making prescriptions “talk” to stroke and heart attack survivors to improve adherence: Results of a randomized clinical trial (The Talking Rx Study) | Kamal et al., 2018 | Included |
| 44. | Fixed Low-Dose Triple Combination Antihypertensive Medication vs Usual Care for Blood Pressure Control in Patients With Mild to Moderate Hypertension in Sri Lanka | Webster et al., 2018 | Excluded: Wrong outcome |
| 45. | Cost-effectiveness of a fixed dose combination (polypill) in secondary prevention of cardiovascular diseases in India: Within-trial cost-effectiveness analysis of the UMPIRE trial | Kavitha singh et al., 2018 | Excluded: Cost effective analysis |
| 46. | Role development of community health workers for cardiovascular disease prevention in India | Khetan et al., 2018 | Excluded: Wrong study design, outcome |
| 47. | Mobile phone text messaging to improve medication adherence in secondary prevention of cardiovascular disease | Adler et al., 2017 | Excluded: Wrong Study design |
| 48. | Impact of multimodal interventions on medication nonadherence among elderly hypertensives: a randomized controlled study | Sheilini *et al.,* 2019 | Included |
| 49. | Enhanced hypertension care through private clinics in Pakistan: a cluster randomised trial. | Khan et al.,2019 | Excluded: Wrong outcome |
| 50. | An Interactive Voice Response and Text Message Intervention to Improve Blood Pressure Control Among Individuals with Hypertension Receiving Care at an Urban Indian Health Organization: Protocol and Baseline Characteristics of a Pragmatic Randomized Controlled Trial | E B Schroeder et al., 2019 | Excluded: Study Protocol |
| 51. | Control of blood pressure and risk attenuation: a public health intervention in rural Bangladesh, Pakistan, and Sri Lanka: feasibility trial results | Jafar et al., 2016 | Included |
| 52. | Community health worker-based intervention for adherence to drugs and lifestyle change after acute coronary syndrome: a multicentre, open, randomised controlled trial | Xavier et al., 2016 | Included |
| 53. | Community-based intervention for blood pressure reduction in Nepal (COBIN trial): a cluster-randomized controlled trial | Neupane *et al.,* 2023 | Included |
| 54. | Process evaluation of the impact and acceptability of a polypill for prevention of cardiovascular disease | Wood F et al.,2015 | Excluded: Wrong study design |
| 55. | Rationale and design of a randomized controlled trial evaluating community health worker-based interventions for the secondary prevention of acute coronary syndromes in India (SPREAD) | Kamath et al.,2014 | Excluded: Wrong outcome |
| 56. | Adherence to cardiovascular medications in the South Asian population: A systematic review of current evidence and future directions | J M Akeroyd et al., 2015 | Excluded: Wrong study design |
| 57. | Cost-Effectiveness of Community-Based Strategies for Blood Pressure Control in a Low-Income Developing Country: Findings from a Cluster-Randomized, Factorial-Controlled Trial | T H Jafar et al., 2011 | Excluded: Wrong study design |
| 58. | Community-Based Interventions to Promote Blood Pressure Control in a Developing Country: A Cluster Randomized Trial | T H Jafar et al., 2009 | Excluded: Wrong outcome |
| 59. | Effect of general practitioner education on adherence to antihypertensive drugs: cluster randomised controlled trial | Qureshi *et al.,* 2007 | Included |
| 60. | Factors precipitating congestive heart failure--role of patient non-compliance | Joshi PP et al., 1999 | Excluded: Wrong study outcome |
| 61. | South Asians living in the UK and adherence to coronary heart disease medication: a mixed- method study | Jalal et al., 2018 | Included |
| 62. | Management of hypertension with the fixed combination of perindopril and amlodipine in daily clinical practice: results from the STRONG prospective, observational, multicenter study | Bahl et al., 2009 | Included |
| 63. | Cardiovascular disease risk management in a primary health care setting of north India | Kar et al., 2008 | Included |
| 64. | Intervention to improve patient adherence with Antihypertensive Medications at a tertiary care teaching hospital | Palanisamy *et al*., 2009 | Included |
| 65. | Assessment of impact of medication counseling on patients' medication knowledge and compliance in an outpatient clinic in South India | Ponnusankar *et al.,* 2004 | Included |
| 66. | A Polypill for primary prevention of cardiovascular disease: a feasibility study of the World Health Organization | Soliman *et al.,* 2011 | Included |
| 67. | Effects of a fixed-dose combination strategy on adherence and risk factors in patients with or at high risk of CVD: the UMPIRE randomized clinical trial | Thom et al., 2013 | Included |
| 68. | South Asians’ cardiac medication adherence | T A Ens et al., 2014 | Excluded: Wrong study design |
