# Supplementary material for: Medication adherence interventions for South Asian populations with cardiovascular disease: A systematic review and narrative synthesis
Source: PLOS Glob Public Health. 2026 Aug 12;6(8):e0006809. doi: 10.1371/journal.pgph.0006809 (PMC13465864; doi:10.1371/journal.pgph.0006809)
Supplement: Supplementary file 4 [file pgph.0006809.s006.docx]

June 2026,

Dear Editor, PLOS Global Public Health

***Manuscript: Medication adherence interventions for South Asian populations with cardiovascular disease: A systematic review and narrative synthesis***

We are grateful to the Editor and Reviewers for their time and thoughtful engagement with our manuscript. We were delighted to receive such encouraging and constructive feedback on our work exploring this important and previously under-researched area.

One Reviewer noted that *“the review was conducted thoroughly and presented well”,* which we found most gratifying.

We have carefully considered each point raised and have revised the manuscript accordingly. Our detailed, point-by-point responses are set out below. We hope that the revised manuscript meets with your approval, and we remain very happy to be contacted should any further clarification be required.

With Kindest regards,

Hiranmayi Madabhushi Hari

On behalf of the co-authors

## **Response to Journal Requirements**

1) **Data Availability Statement**

*The journal requested confirmation that all raw data required to replicate the study findings are available, including values behind means and standard deviations, values used to build graphs, and points extracted from images for analysis.*

**Response:** Thank you for raising this. We wish to clarify that this study is a systematic review with narrative synthesis and did not generate any original quantitative data. As such, requirements relating to means, standard deviations, graph values, and image-extracted data points are not applicable to this submission; instead, they can be found in the original studies as reported by their original authors. However, to support full transparency and replicability, we have now provided the complete data extraction form as a Supplementary file S4, which contains all data extracted from each of the included primary studies.

2) **Copyright and Licensing**

The journal noted potential copyright concerns regarding Figures 2, 3, and 4, including the base layer of maps and icons/clip-art used within the figures.

**Response:** Thank you for drawing our attention to these concerns. We have carefully reviewed all figures and replaced the previous versions with revised versions that comply with PLOS's CC BY 4.0 license requirements. Updated figure legends reflecting these changes have been incorporated into the revised manuscript. The specific steps taken are as follows:

- **Figure 2 (PICO framework used in this study):** Icons have been sourced from Openclipart (<https://openclipart.org/>), which provides content under a CC0 public domain dedication, fully compatible with CC BY 4.0.
- **Figure 3 (Geographic distribution):** The base layer map has been replaced with one from Equal Earth (<https://equal-earth.com/>), which is available in the public domain. The figure legend now includes a direct link to the source and its terms of use.
- **Figure 4 (Seven key thematic areas in this review):** Icons have been sourced from Openclipart (<https://openclipart.org/>).

3) **Supporting Information Legends**

The journal noted the absence of a list of legends for the Supporting Information files.

**Response:** Thank you for this observation. A full list of legends for all Supporting Information files has now been added to the manuscript following the References section.

4) **Data Availability for Systematic Reviews**

*The journal requested a numbered table of all studies identified, a full data extraction table, risk of bias assessments, handling of missing data, and reasons for not conducting a meta-analysis.*

**Response:** Thank you for these requirements. We have addressed each point as follows:

- **Table of all studies identified, including exclusions with reasons:** This is now provided as Supplementary File S3 (*Studies Assessed in Full Text*).
- **Full data extraction table:** All data extracted from the included studies, along with the names of data extractors, dates of extraction, and confirmation of eligibility, are now provided as Supplementary File S4 (*Data Extraction - Included Studies*).
- **Quality/certainty assessments:** These are included in the manuscript.
- **Risk of bias, Missing data and data conversions:** As this is a narrative synthesis, these are not applicable.
- **Reference list:** The reference list has been thoroughly reviewed and updated. All changes are reflected in the tracked-changes version of the manuscript.

## **Response to Reviewer Comments**

**Reviewer 1**

**Comment 1:** *Kindly mention reference to IDF atlas 2025 as diabetes is a cause of CVD mortality and morbidity.*

**Response:** We fully agree that the relationship between diabetes and cardiovascular disease mortality and morbidity is of importance. The IDF Diabetes Atlas 2025 has now been cited and discussed in the relevant section of the revised manuscript (page 34).

**Comment 2:** *Non-availability of FDC in public health systems in middle-income countries is a major constraint; kindly highlight any study related to this.*

**Response:** We agree entirely that the limited availability of fixed-dose combinations (FDC) in public health systems in low- and middle-income countries represents a significant barrier to medication adherence. This issue is now addressed in the revised manuscript (page 37), with reference to relevant studies identified.

**Reviewer 2**

**Comment 1:** *The review was conducted thoroughly and presented well.*

**Response:** We are very grateful to the Reviewer for this kind and encouraging assessment.

**Comment 2:** *My only recommendation concerns the figures: the identification flow diagram appeared blurred. Given the importance of adhering to the PRISMA format, it would strengthen the manuscript to include a clear, high-quality flowchart.*

**Response:** Thank you for noting this. A high-resolution version of the PRISMA 2020 flow diagram has now been produced and resubmitted with this revision.

**Reviewer 3**

**Comment 1:** *Please mention whether the systematic review was registered with PROSPERO and if not, reasons for doing so.*

**Response:** Thank you for raising this important point. As noted on page 5 of the manuscript, this review was not prospectively registered with PROSPERO. We wish to be transparent about the reasons: the literature search and initial screening activities commenced prior to the finalisation of a protocol suitable for PROSPERO submission and PROSPERO has strict regulations for retrospective registration once review activities have begun. To support transparency in the absence of registration, the review was conducted in adherence with the PRISMA 2020 guidelines, using a pre-specified PICO framework and eligibility criteria (Table 1), complete details of which are provided in the Supplementary Files, including the completed PRISMA checklist and search strategy.

**Comments 2 and 3:** *If there are any assumptions about missing or unclear information or any data conversions, that should also be mentioned* and *Reasons for not attempting meta-analysis may also be mentioned.*

**Response:** Thank you for these thoughtful points. We would like to clarify the following:

Regarding **missing data and data conversions**: As this is a systematic review with narrative synthesis, all data presented in this review are derived directly from the original included studies. There were no instances of data conversion or imputation of missing values.

Regarding **the decision not to conduct a meta-analysis**: The 29 included studies displayed considerable heterogeneity across study designs, intervention types, populations, follow-up periods, and adherence measurement tools- the latter encompassing self-report scales (MMAS-8, MMAS-4, MARS, DAI-10, HyCompS, BMQ-S) as well as objective measures (MEMS, pill counts). Outcome reporting formats also varied substantially across studies. Given this degree of heterogeneity, statistical pooling would have risked producing a misleading summary effect; narrative synthesis was therefore the most appropriate and methodologically defensible approach.
